# Expression of Viral Antigen by the Liver Leads to Chronic Infection Through the Generation of Regulatory T Cells

**DOI:** 10.1016/j.jcmgh.2015.02.002

**Published:** 2015-02-24

**Authors:** Pascal Lapierre, Valérie Janelle, Marie-Pierre Langlois, Esther Tarrab, Tania Charpentier, Alain Lamarre

**Affiliations:** Immunovirology laboratory, Institut national de la recherche scientifique, INRS-Institut Armand-Frappier, Laval, Quebec, Canada

**Keywords:** Chronic Infection, Hepatitis, Tolerance, ALT, alanine aminotransferase, APC, allophycocyanin, Arm, Armstrong strain, BTLA, B and T lymphocyte attenuator, CFSE, carboxyfluorescein diacetate succinimidyl ester, CTL, cytotoxic T lymphocyte, ELISA, enzyme-linked immunoassay, FACS, fluorescence-activated cell sorter, FoxP3, forkhead box P3, GP, glycoprotein, HBV, hepatitis B virus, HCV, hepatitis C virus, IFN, interferon, IL, interleukin, IP, intraperitoneal, IV, intravenous, LCMV, lymphocytic choriomeningitis virus, LIL, liver-infiltrating lymphocytes, NP, nucleoprotein, P14, GP_33–41_-specific TCR transgenic, PD-1, programmed death-1, PD-L1, programmed death-ligand-1, PE, phycoerythrin, pfu, plaque-forming units, RAG, recombination-activating gene, TCR, T-cell receptor, TNF-α, tumor necrosis factor-α, TNP4, NP_396–404_-specific TCR transgenic, Treg, regulatory T cell, TTR, transthyretin

## Abstract

**Background & Aims:**

The constant exposure of the liver to food and bacterial antigens through the mesenteric circulation requires it to maintain tolerance while preserving the ability to mount an effective immune response against pathogens. We investigated the contribution of the liver’s tolerogenic nature on the establishment of chronic viral infections.

**Methods:**

TTR-NP mice, which express the nucleoprotein (NP) of lymphocytic choriomeningitis virus (LCMV) specifically in hepatocytes under control of a modified transthyretin (TTR) promoter, were infected with the Armstrong (Arm) or WE acute strains of LCMV.

**Results:**

The infection persisted for at least 147 days in TTR-NP mice. Expression of NP by the liver induced a strong peripheral tolerance against NP that was mediated by interleukin-10-secreting CD4^+^ regulatory T cells, leading to high PD-1 (programmed death-1) expression and reduced effector function of virus-specific T cells. Despite an active immune response against LCMV, peripheral tolerance against a single viral protein was sufficient to induce T-cell exhaustion and chronic LCMV Armstrong (Arm) or WE infection by limiting the antiviral T-cell response in an otherwise immunocompetent host. Regulatory T-cell depletion of chronically infected TTR-NP mice led to functional restoration of LCMV-specific CD4^+^ and CD8^+^ T cell responses and viral clearance.

**Conclusions:**

Expression of a viral antigen by hepatocytes can induce a state of peripheral tolerance mediated by regulatory T cells that can lead to the establishment of a chronic viral infection. Strategies targeting regulatory T cells in patients chronically infected with hepatotropic viruses could represent a promising approach to restore functional antiviral immunity and clear infection.

**Summary**

Expression by the liver of a viral protein induced an immunologic tolerance mediated by interleukin-10-secreting regulatory T cells that impaired the antiviral T-cell response, leading to a chronic infection. This mechanism could be involved in the establishment of persistent infection by hepatotropic viruses.

The liver is an immunoprivileged site prone to tolerance induction. For example, liver grafts are accepted without immunosuppression in several mammals,^1^ and oral tolerance is abrogated when a portacaval shunt is performed.^2^ The liver also has the unique ability among solid organs to activate naive CD8^+^ T lymphocytes in an antigen-specific manner, a process that can be inefficient and lead to apoptosis through a Bim-dependant pathway.^3^

The liver is also host to several chronic infections, but infection of the liver does not inevitably lead to viral persistence. Strong innate immune responses combined with specific T-cell responses can overcome viral escape mechanisms—as in hepatitis A infection or resolved acute hepatitis B (HBV) or C virus (HCV) infections—and achieve viral clearance. However, as observed in 50% to 80% of HCV infections, pathogens can evade early innate and adaptive immune responses through high antigen loads and increased coinhibitory signaling locally in the inflamed liver, leading to T-cell exhaustion and viral persistence.^4^

After exposure, HCV reaches maximal titers several weeks before the induction of detectable humoral or cellular immune responses and the onset of liver disease; in cases where HCV titers remain relatively low, T cell responses may remain undetectable even during chronic infection.^5^ Therefore, the liver may be exposed to HCV antigens in absence of strong immune responses. It has recently been shown in a model of chronic viral infection that CD4^+^ regulatory T-cell depletion in combination with programmed-death-ligand-1 (PD-L1) blockade can significantly reduce viral titers, highlighting the importance of immune inhibitory signals in the outcome of viral infections.^6^ Thus, the liver’s ability to induce tolerance to locally expressed antigens could contribute to the development of chronic liver infections by altering the immunologic response against liver-expressed viral antigens.^7^ Therefore, we directly assessed whether expression of a viral antigen by hepatocytes can induce a state of peripheral tolerance able to contribute to viral persistence.

We show that expression of the nucleoprotein (NP) from lymphocytic choriomeningitis virus (LCMV) specifically in hepatocytes leads to strong peripheral tolerance mediated by interleukin-10 (IL-10)-secreting CD4^+^ forkhead box P3^+^ (FoxP3^+^) regulatory T cells. This allows the establishment of chronic infection by acute strains of LCMV associated with the loss of CD4^+^ and CD8^+^ T-cell effector function, leading to high viral titers in the liver and spleen. Depletion/silencing of CD4^+^ regulatory T cells resulted in the progressive restoration of T-cell function, loss of PD-1 (programmed death-1) expression, and gradual viral clearance. This study demonstrates that expression of a viral antigen in the liver leads to the development of CD4^+^ regulatory T cells able to interfere with the antiviral T-cell response, allowing the establishment of a chronic infection.

## Materials and Methods

### Mice and Viruses

Transthyretin-nucleoprotein (TTR-NP) transgenic mice^8^ (8- to 12-week-old females) expressing LCMV NP specifically in hepatocytes (kindly provided by F. Alvarez, CHU Sainte-Justine, Montreal, Canada) or control C57BL/6 (B6) mice (8- to 12-week-old females) (Charles River, Montreal, Canada) were infected with either acute strains LCMV-Arm (200 plaque-forming units [pfu] intraperitoneally [IP]) or LCMV-WE (200 pfu intravenously [IV]). Hemizygous TTR-NP1/0 mice were generated as a F1 cross between homozygous TTR-NP and C57BL/6 mice. The NP_396–404_-specific T-cell receptor (TCR) transgenic TNP4 mice^9^ were kindly provided by F. Alvarez (CHU Sainte-Justine). The glycoprotein GP_33–41_-specific TCR transgenic P14 mice were kindly provided by P. Ohashi (Princess Margaret Cancer Centre, Toronto, Canada). The recombination-activating gene–nucleoprotein (RAG-NP) mice were obtained by crossing TTR-NP and RAG-1 mice (kindly provided by C Daniel, INRS-Institut Armand-Frappier, Laval, Quebec, Canada). The RAG-1 phenotype was assessed by flow cytometry, and NP expression was monitored by polymerase chain reaction, as previously described elsewhere.^8^

LCMV-WE and Armstrong strains were obtained from R.M. Zinkernagel at the Institute of Experimental Immunology (Zurich, Switzerland). LCMV titration was performed via a standard focus-forming assay. All experiments were performed under protocols approved by the INRS Institutional Committee for Animal Care and following guidelines published by the Canadian Council on Animal Care.

### Lymphocyte Isolation From Liver and Spleen

Livers were perfused via the portal vein with RPMI 1640 (Thermo Fisher Scientific, Mississauga, ON, Canada) and removed. The livers and spleens were finely minced in RPMI 1640, passed through a 100-gauge steel mesh, and centrifuged at 400*g* for 5 minutes at 4°C. Cells contained in the supernatant were washed 3 times with RPMI 1640/5% fetal calf serum before being centrifuged on a Percoll (GE Healthcare Canada, Mississauga, ON, Canada) gradient to purify lymphocytes.^10^

### Flow Cytometry

For the flow cytometry analysis, isolated cells were washed, resuspended in phosphate-buffered saline containing 5% fetal calf serum (fluorescence-activated cell sorter [FACS] buffer), and incubated with directly conjugated primary antibodies for 30 minutes at 4°C. Cells were then washed and resuspended in 200 μL FACS buffer containing 1% formaldehyde. Class I tetramer staining was performed using phycoerythrin (PE)-coupled NP_396–404_ and GP_33–41_ H2-D^b^-restricted tetramers for 30 minutes at 37°C in FACS buffer followed by surface staining. Anti-CD25 allophycocyanin (APC) was purchased from eBioscience (San Diego, CA). Anti-CD45 PE/CF594 was purchased from BD Biosciences (San Jose, CA). Anti-PD-1 fluorescein isothiocyanate and anti-PD-1 allophycocyanin (APC), anti-CD4 APC/Cy7, anti-CD4 fluorescein isothiocyanate, anti-CD8 PE/Cy7, anti-CD62L Alexa Fluor 700, anti-CD44 PercP/Cy5.5, anti-CD3 APC, interferon-γ (IFN-γ) PE, tumor necrosis factor-α (TNF-α) APC, and B and T lymphocyte attenuator (BTLA) Alexa Fluor 647 were purchased from BioLegend (San Diego, CA). Intracellular FoxP3 staining was performed using PE-coupled anti-mouse/rat FoxP3 antibody (clone FJK-16s) and fixation/permeabilization buffer optimized for staining of mouse cells with FJK-16s monoclonal antibodies (eBioscience). Intracellular staining of Helios was performed using Alexa Fluor 647 coupled anti-mouse Helios antibody (clone 22F6) (eBioscience) and using the fixation/permeabilization buffer optimized for staining of mouse cells with FoxP3 (FJK-16s) monoclonal antibodies (eBioscience). Ki-67 protein intracellular staining was performed using anti-mouse/rat Ki-67 efluor450 conjugated antibody (clone SolA15) (eBioscience). Class II tetramer staining (NIH Tetramer Core Facility, Atlanta, GA) (PE-labeled H2-IA^b^ GP_31–45_, GP_66–77_, NP_309–328_, or control H2-IA^b^ hCLIP) was performed at 37°C for 3 hours (2 μg/mL) in FACS buffer. The cells were then washed in FACS buffer, surface stained (CD3, CD4, CD8, CD44, CD62L, CD25, and 7-AAD viability stain) (eBioscience and BioLegend), and fixed. Samples were acquired on a BD LSRFortessa (BD Biosciences) and analyzed using the FlowJo software (Tree Star, Ashland, OR).

### Intracellular Cytokine Staining

Intracellular cytokine staining was performed using isolated lymphocytes stimulated for 5 hours in the presence of 10 U/mL IL-2 and Brefeldin A (10 μg/mL) and one of GP_33–41_, NP_396–404_, GP_61–80_ peptide, or NP_311–325_ (5 μg/mL). Cells were stained for surface and viability markers as described earlier, and then they were fixed and permeabilized for intracellular staining using fixation and permeabilization buffers from BioLegend. Cells were then stained with IFN-γ PE and TNF-α APC (BioLegend). Samples were acquired on a BD LSRFortessa (BD Biosciences) and analyzed using the FlowJo software (Tree Star).

### Type I Interferon and Interleukin-10 Measurement

The IFN-α levels were measured in sera, liver, and spleen homogenates in LCMV-infected B6 and TTR-NP mice with the Verikine mouse IFN-α enzyme-linked immunoassay (ELISA) kit (PBL Interferon Source, Piscataway, NJ) according to the manufacturer’s instructions. IFN-β levels were measured in sera of infected B6 and TTR-NP mice with the Verikine mouse IFN ELISA kit (PBL Interferon Source). The IL-10 serum levels were measured using the multiplex bead immunoassay (Life Technologies/Gibco, Grand Island, NY).

### In Vivo Cytotoxicity Assay

The cytotoxicity assay was performed using carboxyfluorescein diacetate succinimidyl ester (CFSE)-labeled target cells from naive B6 mice (0.2 μM, 1 μM or 5 μM) coated with either the GP_33–41_ or NP_396–404_ peptide (1 μM) or left uncoated (negative control), respectively. These cells (1 × 10^7^ cells each) were injected IV simultaneously in infected or control mice. The mice were killed 4 hours later, and lymphocytes from the spleen and lymph nodes were isolated. Cells were analyzed by flow cytometry on a BD LSRFortessa (BD Biosciences) using the FlowJo software (Tree Star).

### In Vitro Proliferation Assay

Purified CD8^+^ T cells (Stemcell Technologies, Vancouver, BC, Canada) from the spleen and liver were labeled with CFSE (5 μM) (Vybrant CFDA SE Cell Tracer Kit; Molecular Probes, Eugene, OR) and were incubated with syngeneic purified antigen-presenting cells from the spleen,^11^ previously coated with 1 μM of GP_33–41_ or NP_396–404_ peptide, supplemented with 5% fetal calf serum and 30 U/mL of murine recombinant IL-2 (Invitrogen, Carlsbad, CA). After 3 days, cells were labeled with an anti-CD8 antibody (eBioscience) and 7-AAD (Invitrogen). The cells were analyzed by flow cytometry on a BD LSRFortessa (BD Biosciences) and using the FlowJo software (Tree Star).

### In Vivo Proliferation Assay

CD8^+^ T cells were purified from P14 (GP_33–41_-specific TCR) and TNP4 (NP_396–404_-specific TCR) transgenic mice using the EasySep mouse CD8^+^ T-cell enrichment kit (Stemcell Technologies). Purified CD8^+^ T cells (1.5 × 10^6^) from P14 mice were stained with CFSE (5 μM) (Vybrant CFDA SE Cell Tracer Kit; Molecular Probes), and cells from TNP4 mice (1.5 × 10^6^) with Cell Proliferation Dye e450 (10 μM) (eBioscience). We adoptively transferred 1.5 × 10^6^ CD8^+^ T cells from P14 and TNP4 mice by IV route into TTR-NP and B6 control mice. The next day, the mice were infected with 200 pfu of LCMV-Arm IP, and they were killed 4 days later.

Alternatively, 1.5 × 10^6^ CD8^+^ T cells from P14 and TNP4 mice (Stemcell Technologies) were adoptively transferred IV into RAG-NP and *RAG* control mice. The mice were killed 7 days later. Isolated lymphocytes from the spleen and liver were stained (CD3, CD4, CD8, and 7-AAD), and the proliferation of CFSE and e450-labeled cells was analyzed by flow cytometry on a BD LSRFortessa (BD Biosciences) and using the FlowJo software (Tree Star).

### Adoptive T Cell Transfer Into RAG-NP Mice

Splenocytes from B6 mice (1 × 10^8^ cells) were transferred IV into RAG-NP or RAG control mice. After 14 days, mice were killed, and the T cells were surface stained with CD4, CD8, CD25, CD62L, and CD44 (BioLegend) and intracellularly for FoxP3 and Ki-67 (eBioscience).

### Adoptive T Cell Transfer Into TTR-NP

We labeled 2 × 10^6^ purified CD8^+^ T cells from P14 (GP_33–41_) or TNP4 mice (NP_396–404_) (Stemcell Technologies) with CFSE (5 μM) (Vybrant CFDA SE Cell Tracer Kit; Molecular Probes), and they were adoptively transferred IV into TTR-NP or control B6 mice. Two days after transfer, the T cells were isolated from the spleen and liver, and they were analyzed by flow cytometry for proliferation, expression of activation markers (CD25 APC, CD62L Alexa Fluor 700, or CD69 PE) (BioLegend and eBioscience), or intracellular expression of Bim Alexa Fluor 647 (AbD Serotech, Raleigh, NC).

### In Vivo Regulatory CD4^+^CD25^+^ T-Cell Depletion/Silencing

LCMV-infected TTR-NP mice (45 days after infection) were injected IP with 200 μg of anti-CD25 antibodies (PC61.5 clone) or 200 μg of IgG1 isotypic control (BioXcell, West Lebanon, NH) every 7 days. The CD4^+^CD25^+^ regulatory T cell depletion was confirmed by flow cytometry. The mice were killed 28 days after the start of regulatory T cell (Treg) depletion.

### Quantification of LCMV NP Expression Levels by Flow Cytometry

To measure the expression levels of LCMV NP on liver cells, purified anti-NP antibodies from the monoclonal IgG-secreting hybridoma VL4 (kindly given by R.M. Zinkernagel, Institute of Experimental Immunology, Switzerland) were coupled to Alexa Fluor 647 using the Alexa Fluor Protein Labeling Kit (Life Technologies, Rockville, MD). Mechanically disrupted liver cells were separated on a discontinuous 40%/80% Percoll (GE Healthcare Canada) gradient to separate parenchymal from nonparenchymal cells. Parenchymal cells were stained with fluorochrome labeled anti-CD45, 7-AAD, and Alexa647-anti-NP and were analyzed by flow cytometry on a BD LSRFortessa (BD Biosciences) and using FlowJo (Tree Star).

### Serum Alanine Aminotransferase Levels

Serum aminotransferase (ALT) levels were measured using a TRILOGY Multipurpose Analyzer system (DREW Scientific, Dallas, TX).

### Statistical Analysis

Differences between groups were tested using one-way analysis of variance (ANOVA) with Tukey’s post hoc test. The Student *t* test was used when two groups were compared. In all graphs, the error bars represent the standard error of the mean. All statistical analyses were performed using GraphPad Prism version 5 (GraphPad Software, La Jolla, CA).

## Results

### Expression of LCMV Nucleoprotein by Hepatocytes in TTR-NP Mice Leads to Chronic Infection With Acute Strains of LCMV

**Figure 1:**
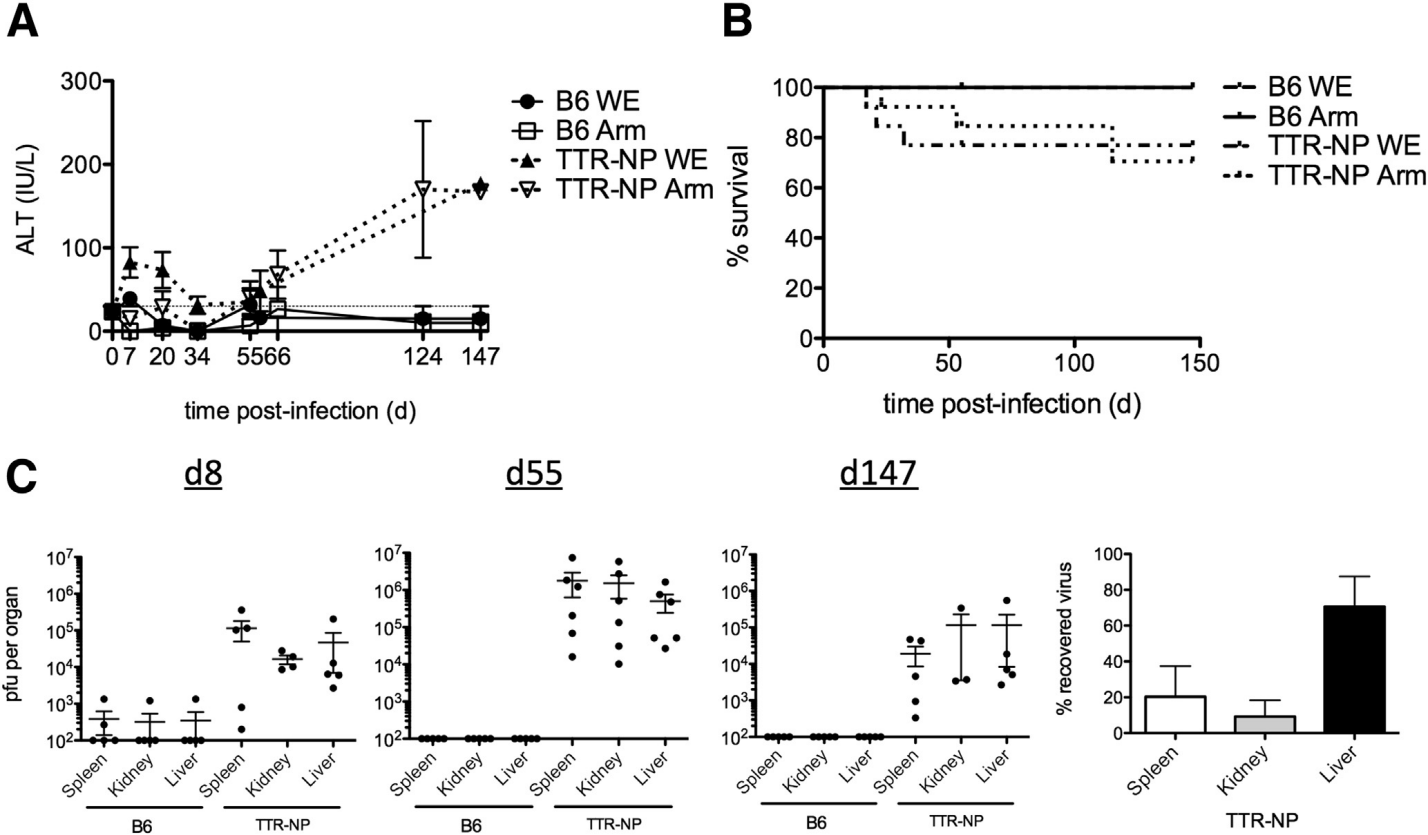
**Transthyretin-nucleoprotein (TTR-NP) mice are chronically infected with acute strains of lymphocytic choriomeningitis virus (LCMV).** (*A*) Alanine aminotransferase (ALT) levels in TTR-NP and B6 mice infected with low-dose LCMV-WE and LCMV-Armstrong (200 plaque-forming units [pfu]). Normal levels are shown (*dotted line*). (*B*) Survival of LCMV-infected TTR-NP and B6 mice. (*C*) LCMV-WE titers in TTR-NP and B6 mice^8^ at 8, 55, and 147 days after infection. Shown is the percentage of recovered virus per organ 147 days after LCMV-WE infection (*right panel*). (Representative data of three independent experiments are shown; n = 4–12 per group.)

TTR-NP mice express LCMV NP specifically in hepatocytes under the control of a modified transthyretin (TTR) promoter.^8^ This liver-specific transthyretin minimal enhancer promoter sequence limits expression to hepatocytes without any detectable expression in the spleen, thymus, muscle, or kidney.^8^ TTR-NP mice were infected with low doses (200 pfu) of LCMV-Arm or WE. Infected mice showed a gradual increase in serum alanine aminotransferase (ALT) levels 55 days after infection indicative of chronic hepatitis (Figure 1*A*). Infected TTR-NP mice showed 20% to 30% mortality rates compared with 0% for infected B6 mice (see Figure 1*B*). The LCMV titers in infected TTR-NP mice were higher on day 8 than in B6 mice and remained high in the spleen, liver, and kidney for over 147 days after infection (see Figure 1*C*). Within each infected mouse, the majority of virus was found in liver (see Figure 1*C*).

### Active Antiviral Immune Response in Infected TTR-NP Mice With High PD-1 Levels on LCMV-Specific CD8^+^ and CD4^+^ T Cells

**Supplementary Figure 1:**
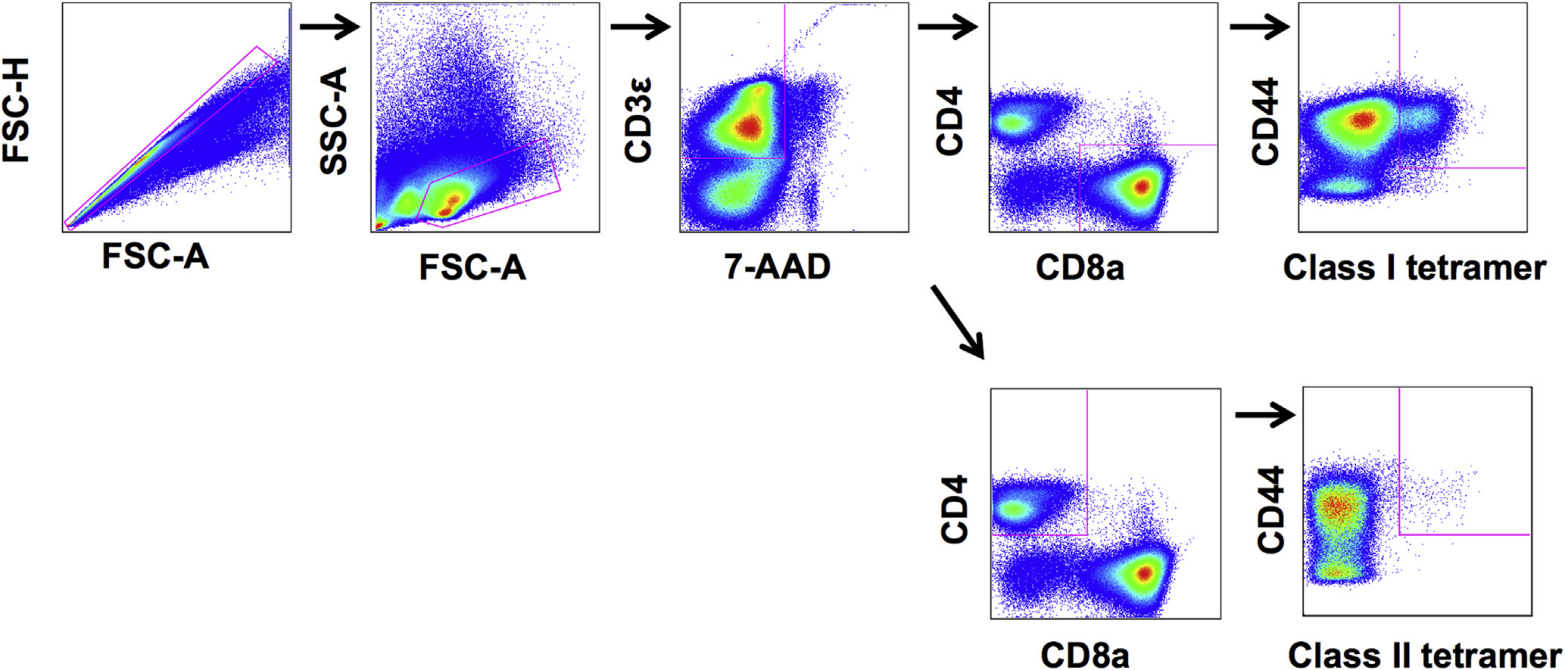
**Flow cytometry gating strategies for class I and II tetramer staining.** Isolated splenocytes (2 × 10^6^ cells) from infected mice were surface stained with anti-mouse antibodies for CD3ε, CD4, CD8, CD44, and appropriate class I (H2-D^b^ NP_396–404_ or GP_33–41_) or class II (H2-IA^b^ NP_309–328_, GP_66–77_, GP_31–45_, or control H2-IA^b^ hCLIP) fluorochrome-labeled tetramers and with fluorescent 7-AAD viability stain. Gating was performed according to these markers to exclude doublets and dead cells and to analyze tetramer staining on either live CD3ε^+^CD4^−^CD8^+^ or CD3ε^+^CD4^+^CD8^−^ lymphocytes.

**Figure 2:**
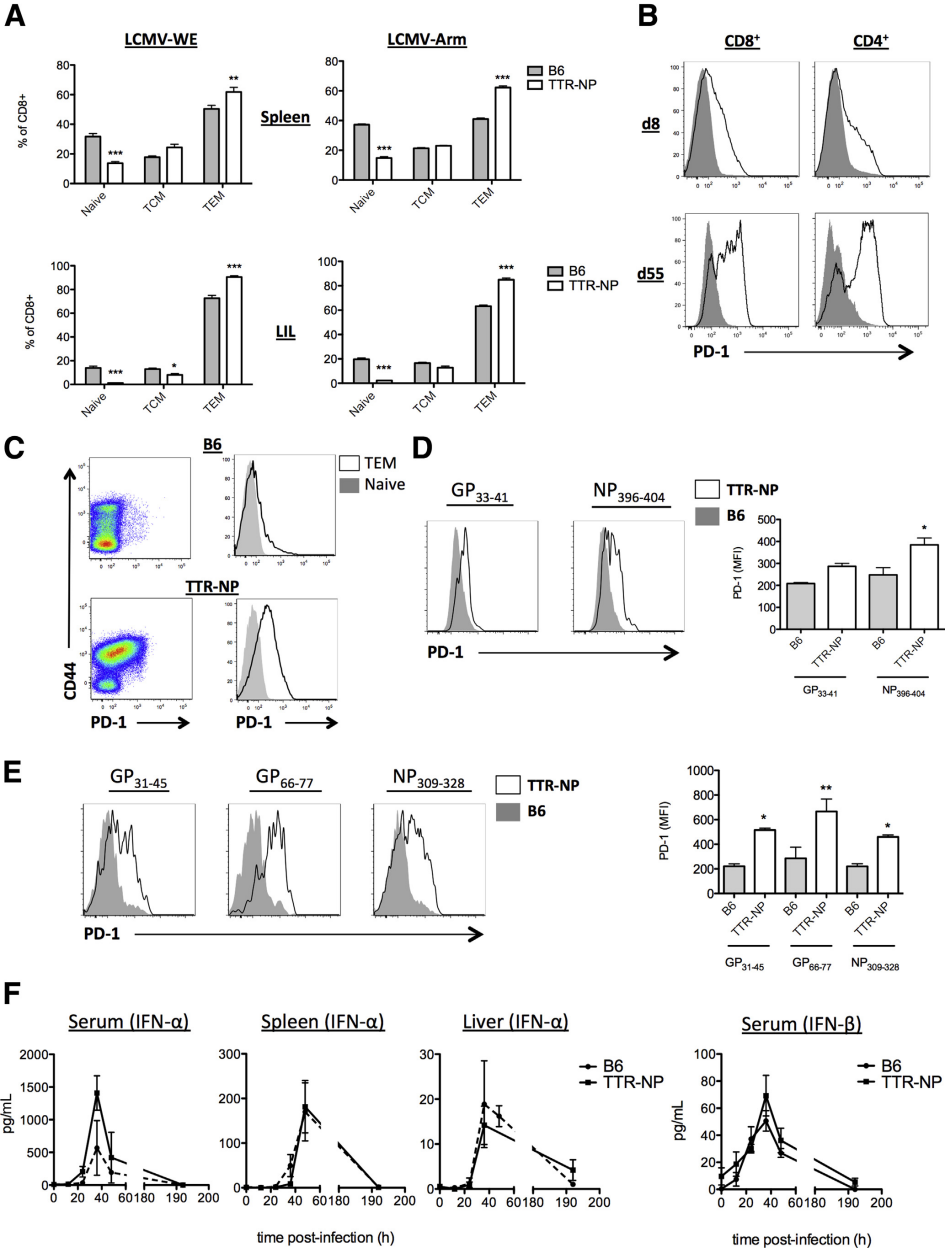
**Lymphocytic choriomeningitis virus (LCMV)-specific CD8**^**+**^**and CD4**^**+**^**T cells from infected transthyretin-nucleoprotein (TTR-NP) mice express high programmed death-1 (PD-1) levels.** (*A*) Levels of CD62L^+^CD44^−^ naive, CD62L^+^CD44^+^ central memory (TCM), and CD62L^−^CD44^+^ effector memory CD8^+^ T cells (TEM) at 55 days after infection. (*B*) Expression of PD-1 by CD8^+^ and CD4^+^ T cells from TTR-NP mice (*dark line*) and B6 mice (*grey shading*) after low-dose lymphocytic choriomeningitis virus (LCMV)-WE infection. (*C*) PD-1 expression on naive and activated/memory CD8^+^ T cells from TTR-NP and B6 mice at 55 days after infection. Expression of PD-1 versus CD44 on CD8^+^ T cells (*left*) and histogram of PD-1 expression on CD44^+^CD62L^−^ effector/memory CD8^+^ T cells (*black line*) and on CD44^−^CD62L^+^ naive CD8^+^ T cells (*grey*). (*D*) Mean fluorescence intensity (MFI) of PD-1 expression on LCMV-specific CD8^+^ T cells from infected TTR-NP and B6 mice 55 days after infection. (*E*) MFI of PD-1 expression on LCMV-specific CD4^+^ T cells from infected TTR-NP and B6 mice 55 days after infection. (*F*) Type 1 interferon levels in serum, spleen, and liver of LCMV-WE-infected mice. (Representative data of two to three independent experiments are shown; n = 4 per group.) *P < .05, **P < .01, ***P < .001.

Fifty-five days after LCMV infection, TTR-NP mice showed higher proportions of effector CD8^+^ T cells in the spleen and among liver-infiltrating lymphocytes (LIL) compared with infected B6 mice (Figure 2*A*). Beginning on day 8 and increasing until day 55 after infection, TTR-NP mice showed high PD-1 expression on CD8^+^ and CD4^+^ T cells (see Figure 2*B*). The PD-1 expression was higher on effector/memory CD8^+^ T cells than on naive CD8^+^ T cells in infected TTR-NP mice (see Figure 2*C*). The levels of PD-1 on epitope-specific CD8^+^ and CD4^+^ T cells were measured using class I and II tetramers (gating strategy described in Supplementary Figure 1). The PD-1 levels were elevated on NP_396–404_ and GP_33–41_ CD8^+^ T cells from TTR-NP mice compared with the B6 mice (see Figure 2*D*). The levels of PD-1 on NP_396–404_-specific CD8^+^ T cells from TTR-NP mice were also significantly higher than those of GP_33–41_ CD8^+^ T cells from TTR-NP mice (see Figure 2*D*). The levels of PD-1 on GP_31–45_, GP_66–77_, and NP_309–328_ CD4^+^ T cells from TTR-NP mice were significantly higher than on those from the infected B6 mice (see Figure 2*E*).

### Normal Type I Interferon Response in LCMV-Infected TTR-NP Mice

Type I IFN is critical for the development of a proper T-cell response against LCMV.^12^ In vitro, LCMV NP has been shown to inhibit the type I IFN response.^13^ The IFN-α and -β levels in the serum, spleen, and liver of infected TTR-NP and B6 mice were compared, and no significant differences were observed (see Figure 2*F*).

### TTR-NP Mice Are Tolerant to LCMV NP

**Figure 3:**
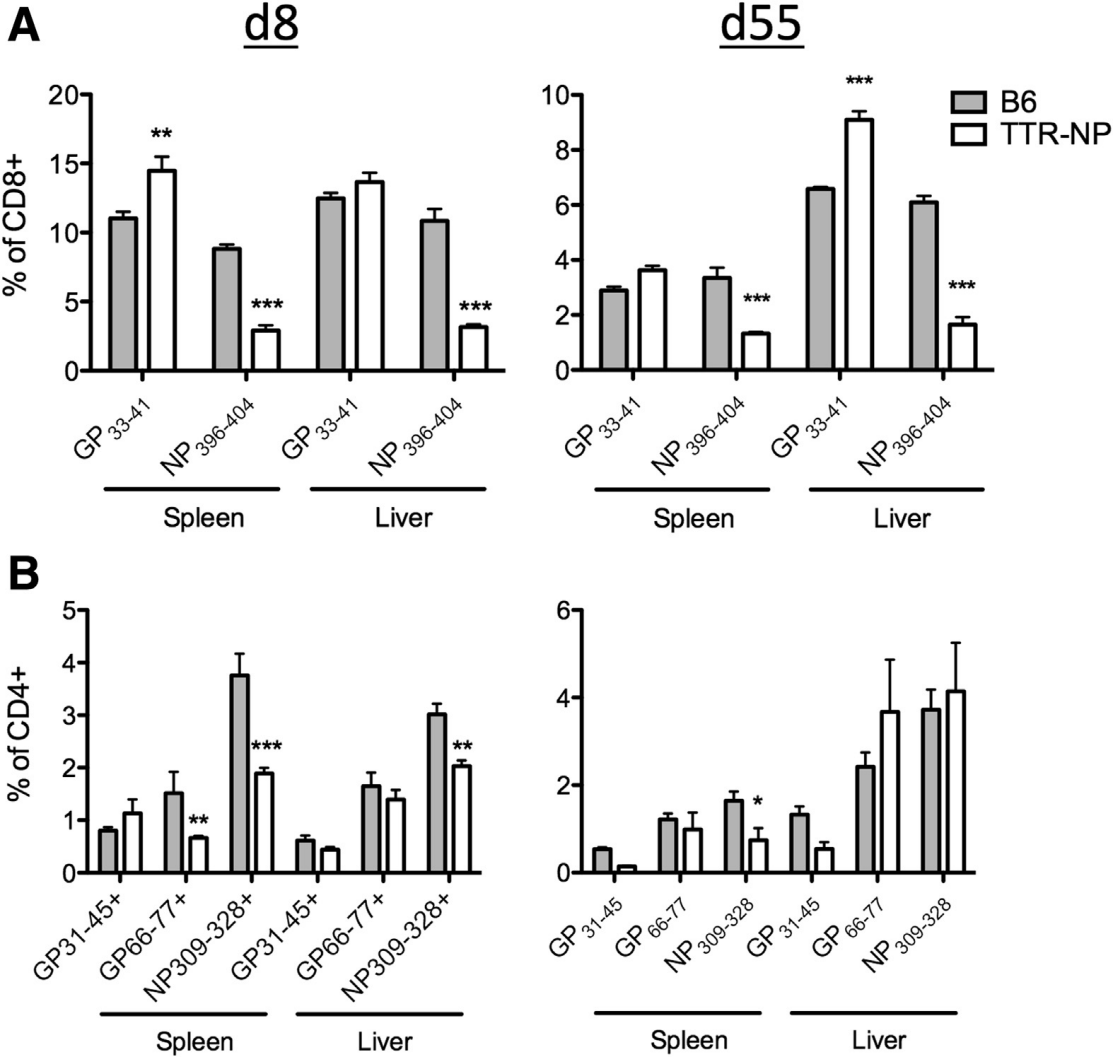
**Lymphocytic choriomeningitis virus (LCMV)-specific CD4**^**+**^**and CD8**^**+**^**T-cell responses in infected transthyretin-nucleoprotein (TTR-NP) mice.** (*A*) Class I tetramer staining (NP_396–404_ and GP_33–41_) of CD8^+^ T cells from spleen and liver of TTR-NP and B6 mice at 8 and 55 days after infection with low-dose (200 plaque-forming units [pfu]) LCMV-WE. (*B*) Class II tetramer staining (IA^b^ NP_309–328_, GP_66–77_, GP_31–45_) of CD4^+^ T cells from the spleen and liver of TTR-NP and B6 mice at 8 and 55 days after infection. Proportions of class I and II tetramer-positive CD8^+^ or CD4^+^ T cells among total CD8^+^ or CD4^+^ T cells are shown. (Representative data of two to three independent experiments are shown; n = 4 per group.) *P < .05, **P < .01, ***P < .001.

**Figure 4:**
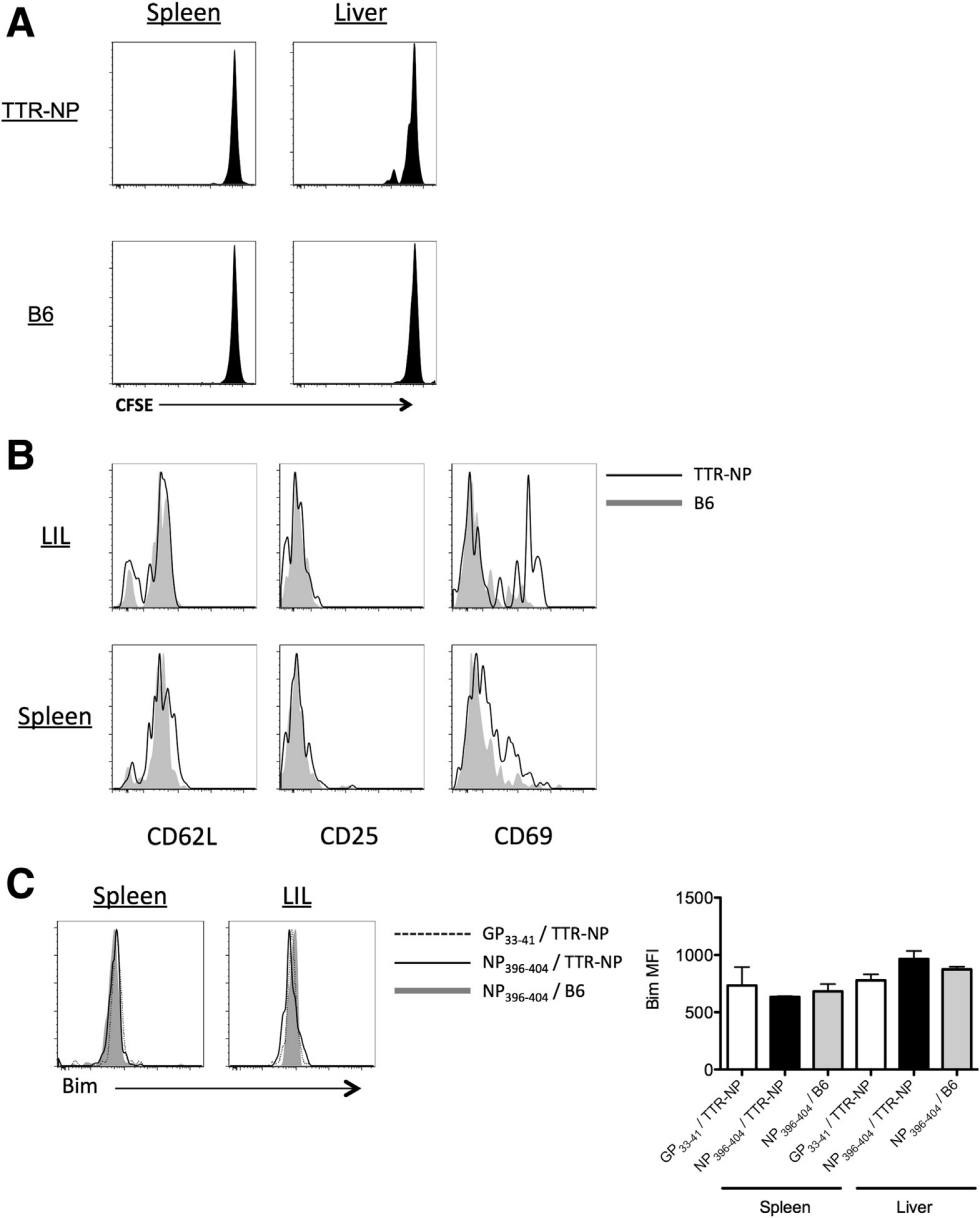
**Hepatocyte-activated nucleoprotein-specific CD8**^**+**^**T cells in uninfected transthyretin-nucleoprotein (TTR-NP) mice do not express proapoptotic Bim protein.** (*A*) 2 × 10^6^ carboxyfluorescein diacetate succinimidyl ester (CFSE)-labeled naive CD8^+^ NP_396–404_-specific T cells isolated from TNP4 mice were transferred IV into uninfected TTR-NP and B6 mice. Two days after the adoptive transfer, the proliferation of CFSE-labeled cells was assessed. (*B*) Expression of activation markers CD62L, CD25, and CD69 was assessed on NP_396–404_-specific T cells 2 days after adoptive transfer in TTR-NP or B6 mice. (*C*) Isolated CFSE-labeled NP_396–404_ and GP_33–41_-specific CD8^+^ T cells from TNP4 and P14 transgenic mice, respectively, were adoptively transferred into TTR-NP and B6 mice and analyzed for Bim expression by flow cytometry 2 days after transfer. (Representative data of two independent experiments are shown; n = 4 per group.)

The TTR-NP mice showed no sign of massive NP-specific clonal deletion, as the CD8^+^ and CD4^+^ T-cell responses against NP were readily detectable by class I and II tetramer staining (Figure 3). Nonetheless, proportions of NP_396–404_-specific CD8^+^ T cells were reduced in both the spleen and liver of TTR-NP mice compared with B6 mice at 8 and 55 days after infection (Figure 3*A*). Eight days after infection, proportions of NP_309–328_-specific CD4^+^ T cells were also reduced in both the spleen and liver of TTR-NP mice compared with B6 mice. Fifty-five days after infection, the levels of NP_309–328_-specific CD4^+^ T cells were reduced in the spleen but similar in the liver (see Figure 3*B*). The levels of GP-specific CD8^+^ (GP_33–41_) and CD4^+^ T cells (GP_31–45_ and GP_66–77_) were similar or higher in TTR-NP mice 8 and 55 days after LCMV infection compared with B6 mice. Primary activation by hepatocytes or liver-resident antigen-presenting cells of naive CD8^+^ T cells can result in a “neglected” phenotype characterized by poor cytotoxic-T lymphocyte (CTL) function, high Bim expression, and cell death.^3^ Adoptive transfer of CFSE-labeled naive NP_396–404_-specific T cells isolated from TNP4 TCR transgenic mice^9^ in uninfected TTR-NP mice led to limited T-cell proliferation, CD69 up-regulation, and a lack of CD25 expression (Figure 4). However, Bim expression levels were not increased (see Figure 4).

**Figure 5:**
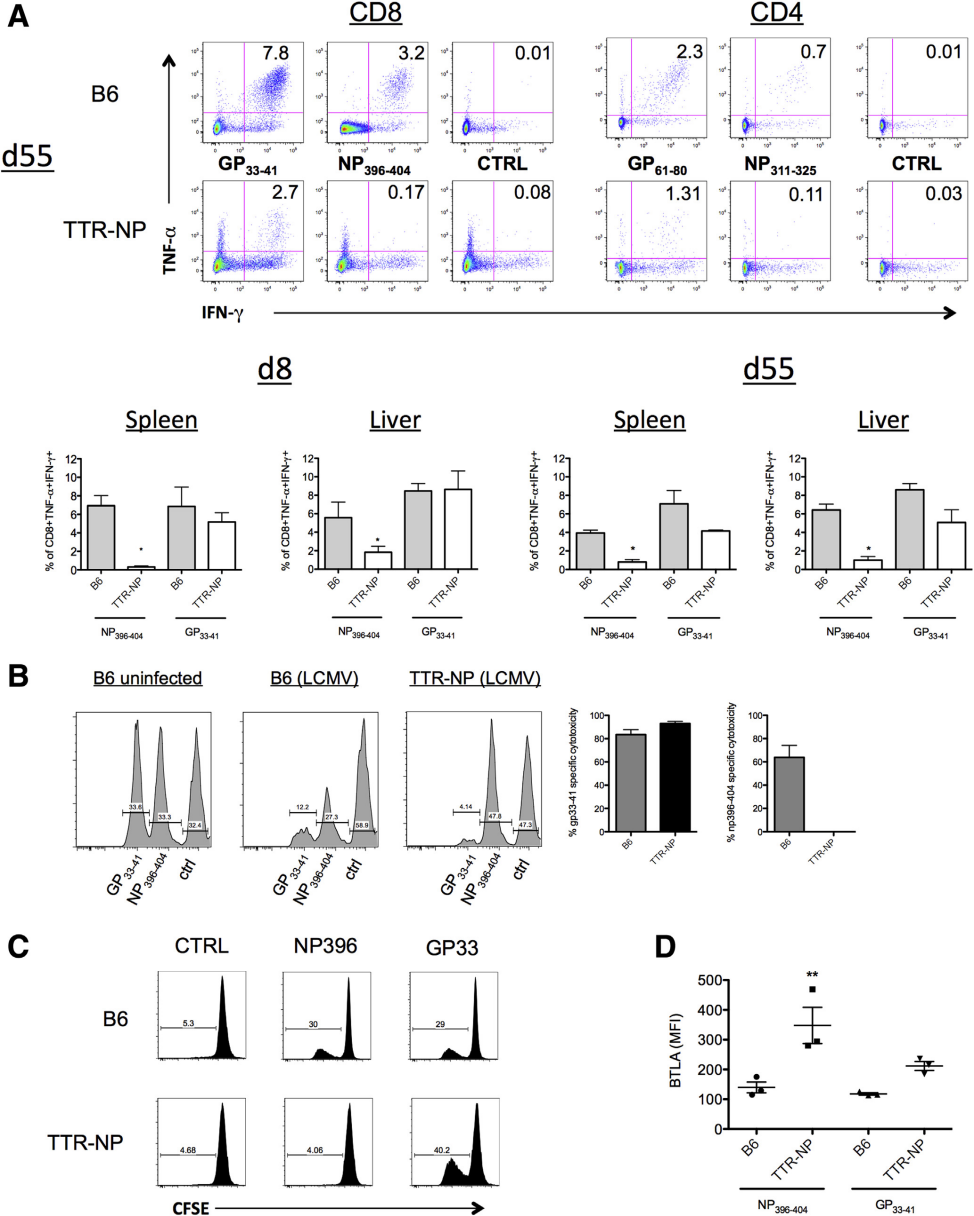
**Transthyretin-nucleoprotein (TTR-NP) mice are tolerant to lymphocytic choriomeningitis virus (LCMV)-NP-derived T-cell epitopes.** (*A*) Proportions of CD8^+^ T cells isolated from infected TTR-NP and B6 mice at 8 and 55 days after LCMV-WE infection secreting both tumor necrosis factor-α (TNF-α) and interferon-γ (IFN-γ) in response to NP_396–404_, GP_33–41_, or control peptide stimulation in vitro. (*B*) In vivo cytotoxicity assay in TTR-NP and B6 mice 30 days after LCMV-WE infection. (*C*) CFSE proliferation assay of CD8^+^ T cells isolated from the spleen of LCMV-WE infected TTR-NP and B6 mice in response to GP_33–41_ or NP_396–404_ T-cell epitopes. (*D*) Expression levels of B and T lymphocyte attenuator (BTLA) on tetramer positive NP_396–404_- and GP_33–41_-specific T cells from TTR-NP and B6 mice at 55 days after LCMV-WE infection. (Representative data of three independent experiments are shown; n = 4 per group.) *P < .05, **P < .01.

These observations are consistent with the fact that although NP396–404-specific T cells are present in fewer numbers in LCMV-infected TTR-NP mice, they are not deleted (see Figure 3). Cytokine-producing LCMV-specific CD8^+^ T cells were reduced in the spleen of TTR-NP mice compared with B6 mice 55 days after infection. Fewer polyfunctional TNF-α^+^ IFN-γ^+^ CD8^+^ T cells were detected after stimulation with GP_33–41_ and very few after stimulation with NP_396–404_ (Figure 5*A*, upper left panel). The CD4^+^ T-cell responses were similarly reduced in TTR-NP mice compared with B6 mice, with very few cells responding to NP_311–325_ peptide stimulation (see Figure 5*A*, upper right panel). Therefore, although TTR-NP mice mount CD4^+^ and CD8^+^ T-cell responses against both NP and GP-derived epitopes after the establishment of chronic LCMV infection (see Figure 3), these cells secrete fewer cytokines 8 days after LCMV infection (see Figure 5*A*, lower left panel), and their levels remained low throughout chronic infection (see Figure 5*A*, lower right panel), especially those targeting NP-derived epitopes.

This reduced number of NP-specific polyfunctional (TNF-α^+^ IFN-γ^+^) T cells could limit the ability of TTR-NP mice to eliminate infected cells and control viral burden. To test this, an in vivo cytotoxicity assay was performed in B6 and TTR-NP mice 30 days after infection. The LCMV-infected B6 mice developed strong CTL responses against both GP_33–41_ and NP_396–404_ pulsed cells (see Figure 5*B*), resulting in their rapid elimination. In TTR-NP mice an efficient CTL response against GP_33–41_-labeled target cells was detected whereas the response against NP_396–404_-pulsed target cells was undetectable (see Figure 5*B*). The in vitro proliferative capacity of these cells was also compromised, as NP_396–404_-specific T cells in TTR-NP mice did not proliferate in response to peptide stimulation whereas GP_33–41_-specific T cells did (see Figure 5*C*).

BTLA is expressed on T cells after antigen-specific induction of anergy in vivo.^14^ BTLA levels were statistically significantly higher on NP_396–404_^+^ CD8^+^ T cells from infected TTR-NP mice than on GP_33–41_^+^ T cells or on those from control B6 mice (see Figure 5*D*).

### Efficient Presentation of Hepatocyte-Expressed NP to CD8^+^ T Cells

**Figure 6:**
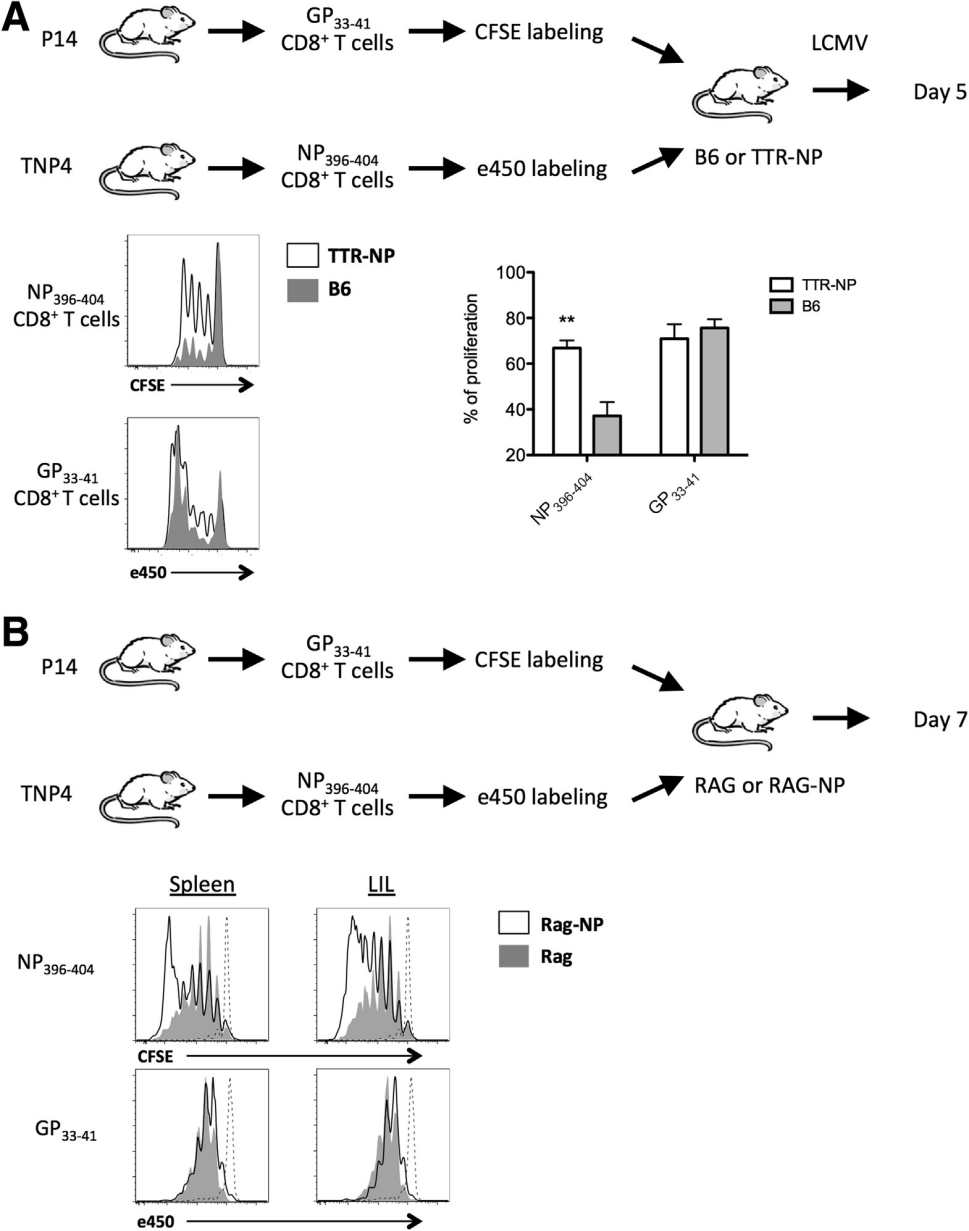
**Hepatocyte-expressed NP is efficiently presented and leads to CD8**^**+**^**T-cell activation.** (*A*) GP_33–41_- and NP_396–404_-specific CD8^+^ T cells isolated from P14 and TNP4 TCR transgenic mice and labeled with CFSE or efluor450, respectively, were adoptively transferred (1.5 × 10^6^ cells) into transthyretin-nucleoprotein (TTR-NP) and B6 mice before low-dose infection with lymphocytic choriomeningitis virus (LCMV)-WE. Proliferation was assessed by flow cytometry 5 days after infection. (*B*) GP_33–41_- and NP_396–404_-specific CD8^+^ T cells were labeled as described and were adoptively transferred into recombination-activating gene (RAG)-deficient or RAG-deficient TTR-NP mice to monitor the proliferation in absence of infection. Proliferation was assessed 7 days later by flow cytometry. The GP_33–41_ (CFSE) and NP_396–404_ specific (efluor450) CD8^+^ T cells before transfer (*dashed lines*) are shown as controls. (Representative data of two independent experiments are shown; n = 4 per group.) **P < .01.

To assess the efficiency of NP presentation early during LCMV infection in TTR-NP mice, GP_33–41_ and NP_396–404_-specific CD8^+^ T cells isolated respectively from P14 and TNP4 TCR transgenic mice were labeled with CFSE or efluor450, respectively, and were adoptively transferred into TTR-NP and B6 mice before LCMV infection. Proliferation of GP_33–41_- and NP_396–404_-specific T cells in the spleen was analyzed 5 days later. The GP_33–41_-specific CD8^+^ T cells proliferated more than the NP_396–404_-specific T cells in B6 mice, highlighting the previously reported immunodominance of this epitope at this early stage of LCMV infection^15^ (Figure 6*A*). However, although the GP_33–41_-specific proliferation was similar between TTR-NP and B6 mice, NP_396–404_-specific proliferation was increased in TTR-NP mice, suggesting that NP_396–404_-T cells are not anergic at early stages of infection (see Figure 6*A*). However, the impact of endogenous versus viral LCMV-NP expression on NP_396–404_-T cell proliferation was indistinguishable in this setting.

To test the hypothesis that endogenous NP in absence of LCMV-expressed NP could lead to the observed NP_396–404_-specific T-cell proliferation, GP_33–41_- and NP_396–404_-specific T cells were adoptively transferred into uninfected RAG-deficient and RAG-deficient TTR-NP mice (RAG-NP). Naive T cells expand in RAG-deficient mice, mostly through an IL-7 driven homeostatic T-cell proliferation process,^16^ thereby facilitating the determination of the proliferation of NP-specific T cells in presence of survival factors (IL-7) without the interference of LCMV-expressed NP. RAG-deficient animals also lack any prior Treg-mediated tolerance to NP that could interfere with NP presentation. Seven days after adoptive transfer, NP_396–404_-specific T-cell proliferation was increased in RAG-NP mice compared with RAG mice whereas GP_33–41_-specific T-cell proliferation was similar (see Figure 6*B*). Therefore, endogenous NP can lead to the proliferation of NP_396–404_-specific T cells in RAG-NP mice.

Taken together these results indicate that although TTR-NP mice are hyporesponsive toward NP after LCMV infection, this does not result from liver-mediated NP_396–404_ CD8^+^ T-cell deletion (see Figure 4) or ineffective T-cell activation through tolerizing endogenous NP presentation (see Figure 6*A* and *B*).

### CD4^+^ FoxP3^+^ Regulatory T Cell Conversion/Expansion in TTR-NP Mice

**Figure 7:**
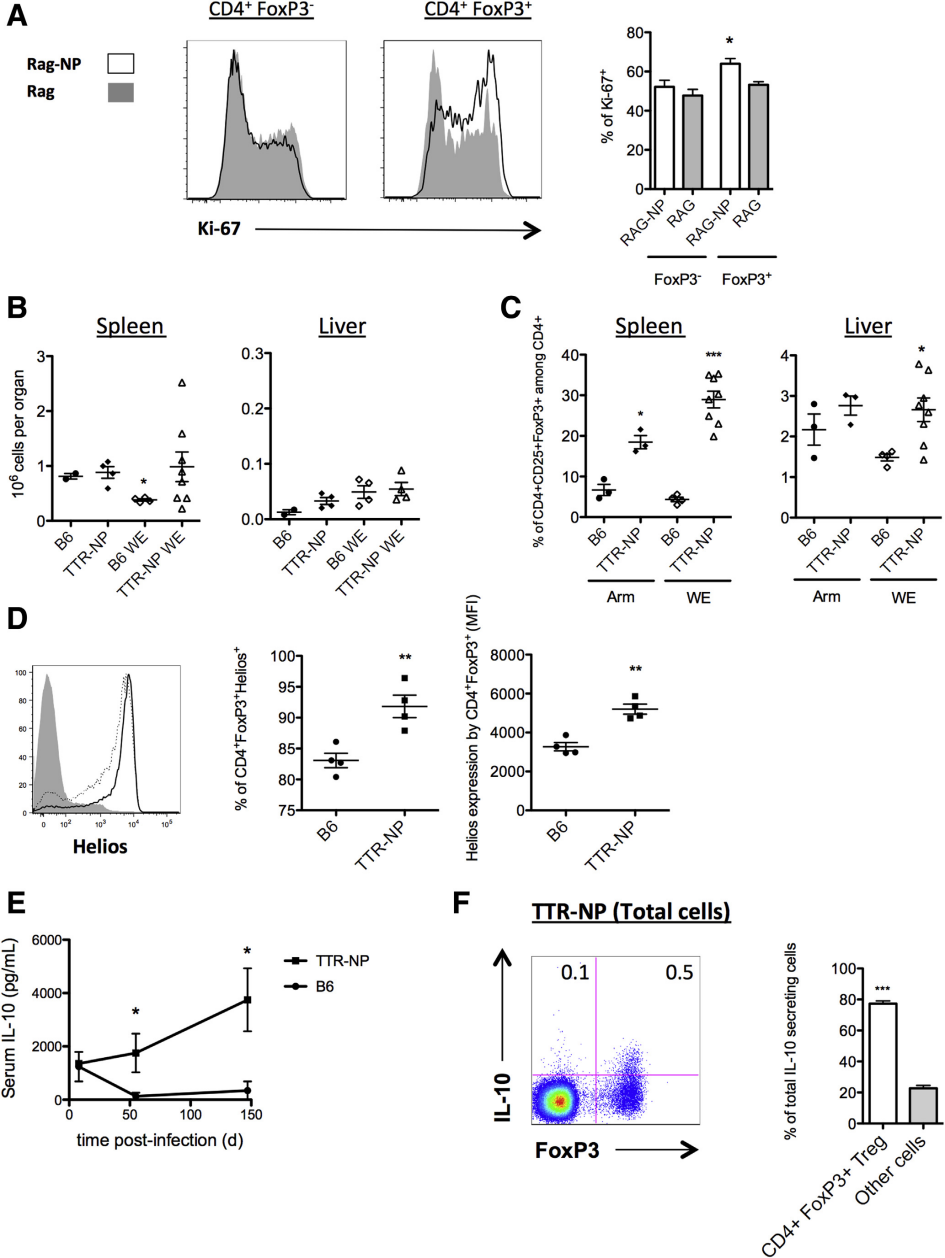
**Interleukin-10 (IL-10)-secreting CD4**^**+**^**FoxP3**^**+**^**regulatory T cells expand after lymphocytic choriomeningitis virus (LCMV) infection of transthyretin-nucleoprotein (TTR-NP) mice.** (*A*) T-cell proliferation of adoptively transferred (1 × 10^8^ T cells from B6 mice) into recombination-activating gene (RAG) and RAG-NP mice monitored by Ki-67 expression 7 days after transfer. (*B*) Number of CD4^+^CD25^+^FoxP3^+^ regulatory T cells in spleen (*left panel*) and liver (*right panel*) before and at 8 days after lymphocytic choriomeningitis virus (LCMV)-WE infection. (*C*) Proportions of CD4^+^CD25^+^FoxP3^+^ regulatory T cells in the spleen (*left panel*) and liver (*right panel*) at 55 days after infection. (*D*) Expression levels of Helios on CD4^+^CD25^+^FoxP3^+^ regulatory T cells in infected TTR-NP (*solid line*) and B6 mice (*dotted line*) at 55 days after LCMV-WE infection. (*E*) Serum levels of IL-10 in infected TTR-NP and B6 mice 8 to 147 days after LCMV-WE infection. (*F*) Secretion of IL-10 by FoxP3^+^-expressing cells among total splenocytes from TTR-NP mice at 55 days after LCMV-WE infection (*left*), and the proportion of IL-10 secreted by CD4^+^FoxP3^+^ regulatory T cells in infected TTR-NP mice (*right*). (Representative data of two to three independent experiments are shown; n = 4–8 per group.) *P < .05, **P < .01, ***P < .001.

To determine whether Tregs could specifically convert/expand in response to liver-expressed NP, 1 × 10^8^ splenocytes from B6 mice were adoptively transferred into RAG and RAG-NP mice, and the T-cell proliferation was monitored by Ki-67 staining. This number of adoptively transferred cells in a RAG-deficient mouse, while minimizing homeostatic proliferation, facilitated tracking of proliferation based solely on NP expression and was not influenced by preestablished tolerance to NP. The CD4^+^ FoxP3^+^ cells, but not the CD4^+^ FoxP3^−^ T cells, showed an increased Ki-67 expression in RAG-NP mice in comparison with RAG mice (Figure 7*A*).

### CD4^+^ Regulatory T Cell Expansion in Infected TTR-NP Mice

The number of CD4^+^ FoxP3^+^ regulatory T cells before LCMV infection was not statistically significantly different between the TTR-NP and B6 mice in the spleen and liver (see Figure 7*B*). Eight days after LCMV-WE infection, the number of splenic Tregs in B6 mice decreased slightly while their numbers remained elevated in infected TTR-NP mice (see Figure 7*B*). The number of liver Tregs was low and did not significantly vary after infection (Figure 7*B*).

Similar results were obtained after LCMV-Arm infection. The TTR-NP mice showed a significant increase in the proportion of CD4^+^ FoxP3^+^ T cells among the CD4^+^ T cells after infection with LCMV-WE or LCMV-Arm compared with the B6 mice in both the spleen and liver (see Figure 7*C*), and they remained elevated throughout the chronic infection. The FoxP3^+^ regulatory T cells from infected TTR-NP mice expressed statistically significantly higher levels of Helios than those from infected B6 mice (see Figure 7*D*). Fifty-five days after infection, the serum levels of IL-10 were statistically significantly higher in the infected TTR-NP mice than in the B6 mice and remained elevated up to 147 days after infection (see Figure 7*E*). In these mice, CD4^+^FoxP3^+^ regulatory T cells were responsible for the majority of IL-10 production among splenocytes (see Figure 7*F*).

### Level of NP Expression in Hepatocytes Is Critical for the Establishment of LCMV Chronicity

**Figure 8:**
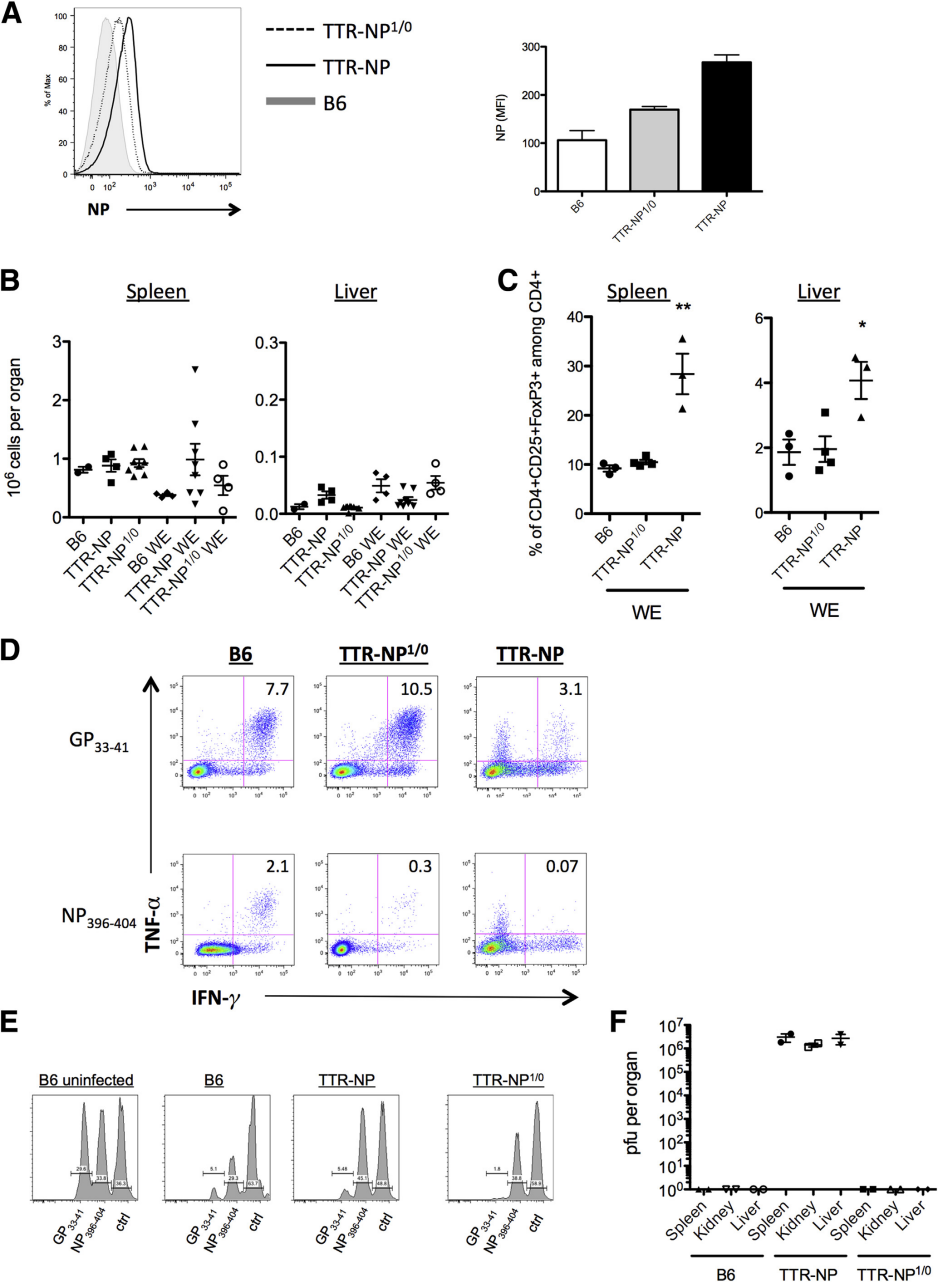
**Hemizygous transthyretin-nucleoprotein (TTR-NP)1/0 (TTR-NP^1/0^) mice clear a lymphocytic choriomeningitis virus (LCMV) infection.** (*A*) Expression levels of LCMV-NP in hepatocytes from hemizygous TTR-NP^1/0^ mice as determined by flow cytometry using fluorochrome-labeled NP-specific antibodies. (*B*) Number of CD4^+^CD25^+^FoxP3^+^ regulatory T cells before infection in hemizygous TTR-NP^1/0^, TTR-NP, and B6 mice as determined by flow cytometry. (*C*) Proportion of CD4^+^CD25^+^FoxP3^+^ regulatory T cells after LCMV-WE infection in hemizygous TTR-NP^1/0^, TTR-NP, and B6 mice at 55 days after infection. (*D*) Proportion of IFN-γ^+^ TNF-α^+^ CD8^+^ T cells from hemizygous TTR-NP^1/0^, TTR-NP, and B6 mice after stimulation with GP_33–41_ or NP_396–404_ LCMV epitopes. (*E*) In vivo cytotoxicity assay in hemizygous TTR-NP^1/0^, TTR-NP, and B6 mice after LCMV-WE infection at 30 days after infection. An in vivo cytotoxicity assay was performed using adoptive transfer of peptide pulsed (GP_33–41_, NP_396–404_, or negative control) CFSE-labeled splenocytes target cells in LCMV-infected and noninfected mice. Cells were isolated and analyzed 4 hours after transfer. (*F*) LCMV-WE titers 30 days after infection in hemizygous TTR-NP^1/0^, TTR-NP, and B6 mice. (Representative data of three independent experiments are shown; n = 4 per group.) *P < .05, **P < .01.

The expression levels of NP in the liver of TTR-NP mice could have a direct impact on the fate of CD8^+^ T cells^17^ and the chronicity of infection. To assess this, hemizygous TTR-NP^1/0^ mice expressing NP at half the levels of that of TTR-NP mice were used (Figure 8*A*). The number of CD4^+^ regulatory T cells before infection was similar between TTR-NP^1/0^ and TTR-NP mice in both the spleen and liver (see Figure 8*B*). However, the significant expansion of CD4^+^ FoxP3^+^ Tregs observed in the TTR-NP mice after LCMV infection was not seen in the hemizygous TTR-NP^1/0^ mice (see Figure 8*C*). LCMV infection of hemizygous TTR-NP^1/0^ mice led to increased IFN-γ and TNF-α CD8^+^ T cell responses against both GP_33–41_ and NP_396–404_ epitopes compared with the TTR-NP mice. However, the NP_396–404_ T cell responses did not reach that of infected B6 mice (see Figure 8*D*).

T cells from hemizygous TTR-NP^1/0^ mice showed increased in vivo cytotoxicity against NP-expressing target cells compared with LCMV-infected TTR-NP mice while remaining lower than that of B6 mice (see Figure 8*E*). Despite the impaired T-cell response against NP-derived epitopes in hemizygous TTR-NP^1/0^ mice, infection with acute strains of LCMV did not persist (see Figure 8*F*).

### Depletion/Silencing of CD4^+^ Regulatory T Cells Leads to Viral Clearance in Chronically Infected TTR-NP Mice

**Figure 9:**
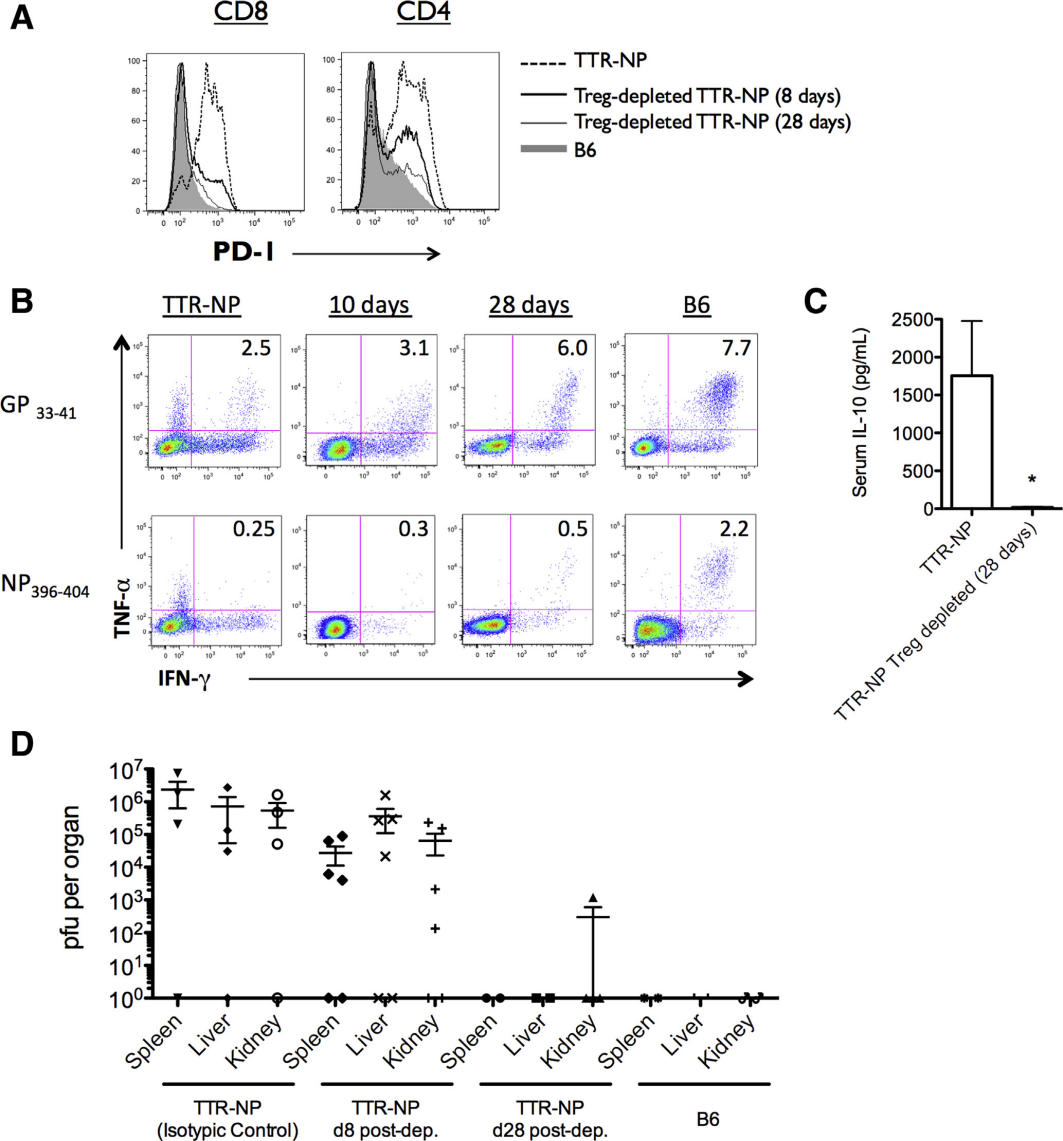
**CD4**^**+**^**regulatory T cell (Treg) depletion/silencing leads to viral clearance.** (*A*) Programmed death-1 (PD-1) expression levels on T cells 8 and 28 days after anti-CD25-mediated Treg depletion performed 45 days after infection. (*B*) Proportions of lymphocytic choriomeningitis virus (LCMV)-specific interferonγ^+^ (IFN-γ^+^) tumor necrosis factor-α^+^ (TNF-α^+^) CD8^+^ T cells from LCMV-WE-infected transthyretin-nucleoprotein (TTR-NP) mice at 10 and 28 days after Treg depletion. (*C*) Interleukin-10 (IL-10) serum levels in LCMV-WE-infected TTR-NP mice after Treg depletion. (*D*) LCMV titers at 8 and 28 days after Treg depletion in LCMV-infected TTR-NP mice. (Representative data of three independent experiments are shown; n = 4 per group.) *P < .05.

To ascertain the role of CD4^+^ regulatory T cells in viral persistence of infected TTR-NP mice, these cells were depleted/silenced using CD25-specific antibodies. Forty-five days after LCMV infection, the TTR-NP mice were injected weekly with anti-CD25 antibodies (PC61.5) or isotypic control. Treatment reduced the levels of CD4^+^CD25^+^FoxP3^+^ T cells by 96.8% ± 2.1% in the spleen and 87.6% ± 1.6% in the liver. Over a 28-day period, the PD-1 expression by CD4^+^ and CD8^+^ T cells gradually returned to control levels (Figure 9*A*). CD8 T-cell functionality was also improved, with increased numbers of IFN-γ^+^ TNF-α^+^ CD8^+^ T cells after stimulation with GP_33–41_ and NP_396–404_ peptides (see Figure 9*B*).

The serum levels of IL-10 were significantly reduced in infected Treg-depleted/silenced TTR-NP mice compared with infected TTR-NP mice (see Figure 9*C*). LCMV viremia gradually declined, and the mice achieved viral clearance 28 days after beginning the anti-CD25 treatment (see Figure 9*D*).

## Discussion

We found that expression of a viral protein by the liver in the absence of inflammation can establish a state of immunologic tolerance against the virus, leading to the establishment of a persistent infection. This expression led to the generation of regulatory T cells able to impede the antiviral immune response, allowing acute LCMV strains to persist with characteristics normally only observed during chronic LCMV Clone 13 infection, such as T-cell exhaustion and PD-1 up-regulation.^18^ Chronic infection with acute LCMV-Arm or LCMV-WE strains has only been shown to occur in either neonates^19^ or severely immunocompromised animal models such as perforin-invalidated mice.^20^ However, TTR-NP mice are not immunodeficient and mount efficient antiviral T- and B-cell responses against vesicular stomatitis virus and clear infection (data not shown).

TTR-NP mice also develop an early type I interferon response to LCMV similar to that of B6 mice. In vitro experiments have shown that NP expression could inhibit the type I interferon response.^13^ However, NP expression is restricted to the hepatocytes in TTR-NP mice, and these cells are not major producers of type I interferon during LCMV infection.^21^

TTR-NP transgenic mice express NP specifically in hepatocytes under the control of a modified TTR promoter and do not express NP in the thymus.^8^ As evidenced by the presence of NP-specific T cells after infection, extensive central deletion of NP-reactive T lymphocytes does not occur in these mice. This contrasts with a previous transgenic model tolerant to NP due to clonal deletion.^22^ The active peripheral tolerance against NP in TTR-NP mice likely stems from an expansion of or conversion to regulatory CD4^+^ T cells.

FoxP3^+^ regulatory T cells are central to peripheral tolerance both by their direct action on T cells but also by keeping dendritic cells in an immature state that can then efficiently induce peripheral tolerance.^23^ This regulatory T cell-mediated active tolerance curbed the antiviral response against NP-derived epitopes but also interfered with CD4^+^ and CD8^+^ T-cell responses against GP-derived epitopes. CD4^+^ regulatory T cells initially require TCR stimulation to expand and initiate their suppressive effects, but once this condition has been met, their ensuing suppression can act in a non-antigen-specific way, in part through IL-2 sequestration.^24^ Therefore, expansion of CD4^+^ regulatory T cells at high levels after LCMV infection of TTR-NP mice resulted in both antigen-specific and antigen-unspecific suppression of anti-LCMV responses, as seen by the lower numbers of polyfunctional GP- and NP-specific CD4^+^ and CD8^+^ T cells in chronically infected TTR-NP mice.

Interestingly, there is an increased level of GP_33–41_-specific CD8^+^ T cells in the liver compared with the spleen at 55 days after LCMV infection. This suggests a greater retention of these cells in the liver as opposed to the spleen where GP_33–41_-specific CD8^+^ T cell levels decline significantly compared with the levels found at day 8. This increased retention likely stems from a combination of high local viral burden and an inability of NP_396–404_ T cells to exert an efficient cytotoxic response, leading to a shift of T-cell immunodominance in latter stages of infection in these animals.

The expression levels of NP in TTR-NP mice also contributed to the observed LCMV persistence because hemizygous TTR-NP^1/0^ mice did not become chronically infected after LCMV infection. Recently, Tay et al^17^ suggested that low expression levels of a liver-antigen led to the activation of CD8^+^ T cells with full effector functions whereas high levels induced functional exhaustion. This suggests that high NP expression in TTR-NP mice may have led to exhaustion of NP-specific CD8^+^ T cells and could explain why the CD4^+^ regulatory T-cell silencing/depletion of infected TTR-NP, though leading to improved CD8^+^ T cell responses and viral clearance, did not restore CD8^+^ T-cell functionality up to B6 levels.

In addition to their classic roles of maintaining immunologic tolerance to self and preventing autoimmunity, CD4^+^ regulatory T cells can also inhibit immune responses against pathogens. In HCV infection, the increased Treg numbers correlate with higher viral burdens and increased disease activity,^25^ and progression to persistent infection correlates with an expansion of Gal-9-expressing regulatory T cells.^26^ In nonhuman primates, subinfectious exposure to HCV suppressed the T-cell responses against subsequent HCV challenge through the generation of regulatory T cells.^27^ Therefore, although the NP expression in transgenic TTR-NP mice is artificial, expression of HCV viral proteins in presence of low inflammation levels occurs and can lead to the generation of regulatory T cells able to hinder the antiviral immune response.

Regulatory T cells in TTR-NP mice express the Helios transcription factor at higher levels than in B6 mice. Although initially believed to distinguish natural Tregs from peripherally induced Tregs, the expression of the Helios transcription factor has been recently reported in both subsets^28^ and is now believed to reflect the context of stimulation during FoxP3 induction.^29^ These observations preclude using Helios as a marker of thymic Tregs, but its expression is indicative of recent antigenic stimulation,^29^ suggesting that regulatory T cells of infected TTR-NP mice might be more responsive to viral and/or endogenous NP than those of B6 mice.

CD4^+^ FoxP3^+^ regulatory T cells actively secreted IL-10, and its levels dramatically decreased after Treg depletion, leading to viral clearance. Interestingly, IL-10 promoter polymorphisms can predict the initial response to IFN-α treatment of HCV patients, with high producers of IL-10 responding poorly to treatment.^30^ Moreover, early IL-10 predominant responses have been associated with an increased progression to chronic HCV infection in injecting drug users.^31^

Infection with acute LCMV strains is not associated with T-cell exhaustion.^32^ In TTR-NP mice, expression of the T-cell exhaustion marker PD-1 on CD4^+^ and CD8^+^ T cells correlated directly with LCMV titers after Treg depletion and viral clearance (Pearson’s correlation, R^2^ = 0.86, *P* = .008, and R^2^ = 0.88, *P* = .0006, respectively), suggesting that PD-1 expression by T cells is likely secondary to chronic infection and high antigen levels. Thus, the PD-1 expression is triggered by TCR signaling; so long as LCMV persists, PD-1 expression on LCMV-specific T cells will be sustained.^33^ As a result, we speculate that Treg-mediated tolerance against one viral antigen (NP) significantly impaired antiviral immune responses, leading to sustained viral loads and high PD-1 expression levels on T cells. This would explain why PD-1 expression is high on both GP and NP-specific T cells after establishment of chronic LCMV infection and decreases upon viral clearance after Treg depletion. This is in agreement with recent findings by Penaloza-MacMaster et al^6^ that showed significantly reduced viral titers upon CD4^+^ regulatory T-cell depletion and PD-L1 blockade in mice infected with the chronic LCMV strain Clone 13.

Anti-CD25 antibody-mediated silencing/depletion of Tregs in infected TTR-NP mice led to improved T-cell functionality and viral clearance. Anti-CD25 antibody-mediated silencing/depletion of Tregs was chosen because LCMV chronicity requires high NP expression, which would be difficult to achieve using an alternative method such as depletion-inducible Foxp3DTR on a TTR-NP background.

In addition, the use of anti-CD25 has the benefit of potentially being translated more rapidly to clinical application because daclizumab, an anti-CD25 humanized antibody, is already approved for human use.^34^ Daclizumab is used to treat acute-graft rejection by targeting CD25-expressing effector T cells,^34^ but it has also been shown to effectively deplete CD4^+^ regulatory T cells in humans, improving immune responses after tumor-antigen vaccination in patients with metastatic breast cancer.^35^

In conclusion, our results show that expression of a viral protein by hepatocytes can induce a state of active peripheral tolerance, mediated by IL-10-expressing Tregs, sufficient to limit an antiviral T-cell response and lead to the establishment of a chronic viral infection, which suggests that interventions aimed at depleting or silencing Tregs could be useful for the treatment of patients chronically infected with hepatotropic viruses.
